# Supplementary material for: A data-consistent model of the last glaciation in the Alps achieved with physics-driven AI
Source: Nat Commun. 2025 Jan 20;16:848. doi: 10.1038/s41467-025-56168-3 (PMC11747445; doi:10.1038/s41467-025-56168-3)
Supplement: Supplementary file 1 — Supplementary information [file 41467_2025_56168_MOESM1_ESM.pdf]

A data-consistent model of the last glaciation in the Alps achieved with physics-driven AI

Author list

Tancrède P.M. Leger[1,2*+], Guillaume Jouvet[1+], Sarah Kamleitner[1,3], Jürgen Mey[4], Frederic Herman[1], Brandon D. Finley[1], Susan Ivy-Ochs[5], Andreas Vieli[3], Andreas Henz[3], Samuel U. Nussbaumer[3]

[1]Institute of Earth Surface Dynamics, University of Lausanne, Lausanne, Switzerland
[2]Department of Geography, University of Sheffield, Sheffield, United Kingdom
[3]Department of Geography, University of Zurich, Zurich, Switzerland
[4]Institute of Environmental Science and Geography, University of Potsdam, Potsdam, Germany
[5]Laboratory of Ion Beam Physics, ETH Zurich, Zurich, Switzerland

+ These authors contributed equally

* Correspondence and requests for materials should be addressed to T.P.M.L. (email: tancrede.leger@unil.ch)

Supplementary information

Supplementary Table 1: The 10 ensemble-varying parameters and their range values*

Supplementary Table 1. Ensemble-varying parameter ranges

**Varying model parameter**	**System component**	**Range** [min - max]	**Unit**
Pseudo-plastic sliding law U threshold	Sliding	[100 - 2000]	n/a
Topographic control on yield Stress: lower bed elevation treshold	Bed softness	[-500 - 100]	m a.s.l.
Topographic control on yield Stress: upper bed elevation treshold	Bed softness	[2400 - 3000]	m a.s.l.
Flow law enhancement factor	Ice properties	[0.5 - 2]	n/a
Positive Degree Day melt factor for ice	Surface Mass Balance	[6 - 9]	mm *we* $.d^{-1}.°C^{-1}$
Positive Degree Day refreezing factor	Surface Mass Balance	[0.5 - 0.7]	scalar multiplier
Surface air-to-ice temperature offset	Ice enthalpy	[1 - 3]	°C
Basal topography (with or without valley-fill sediments)	Topography	[without sediments - with sediments]	n/a
Catchment-specific precipitation offsets	Climate	[0.85 - 1.15]	scalar multiplier of offset map
Lithospheric elastic thickness	Glacial Isostatic Adjustment	[35000 - 50000]	m

*The initial ranges of parameter values that are sampled from (by the Latin Hypercube algorithm) are selected to be a conservative range (thus exploring quite widely the possible model responses) bracketing values found to best represent the conditions of the AIF during the LGM, as found by previous modelling efforts, including the previous work of Mey et al. (2016), Seguinot et al. (2018), Jouvet et al. (2023). For ensemble-varying parameters that are specific to IGM and this experiment (i.e. the catchment-specific precipitation offset parameter, and topographic control on yield stress parameters), initial ranges were obtained by conducting a series of sensitivity tests prior to running the ensemble and targeting ranges that bracket values shown to produce a better model data fit.

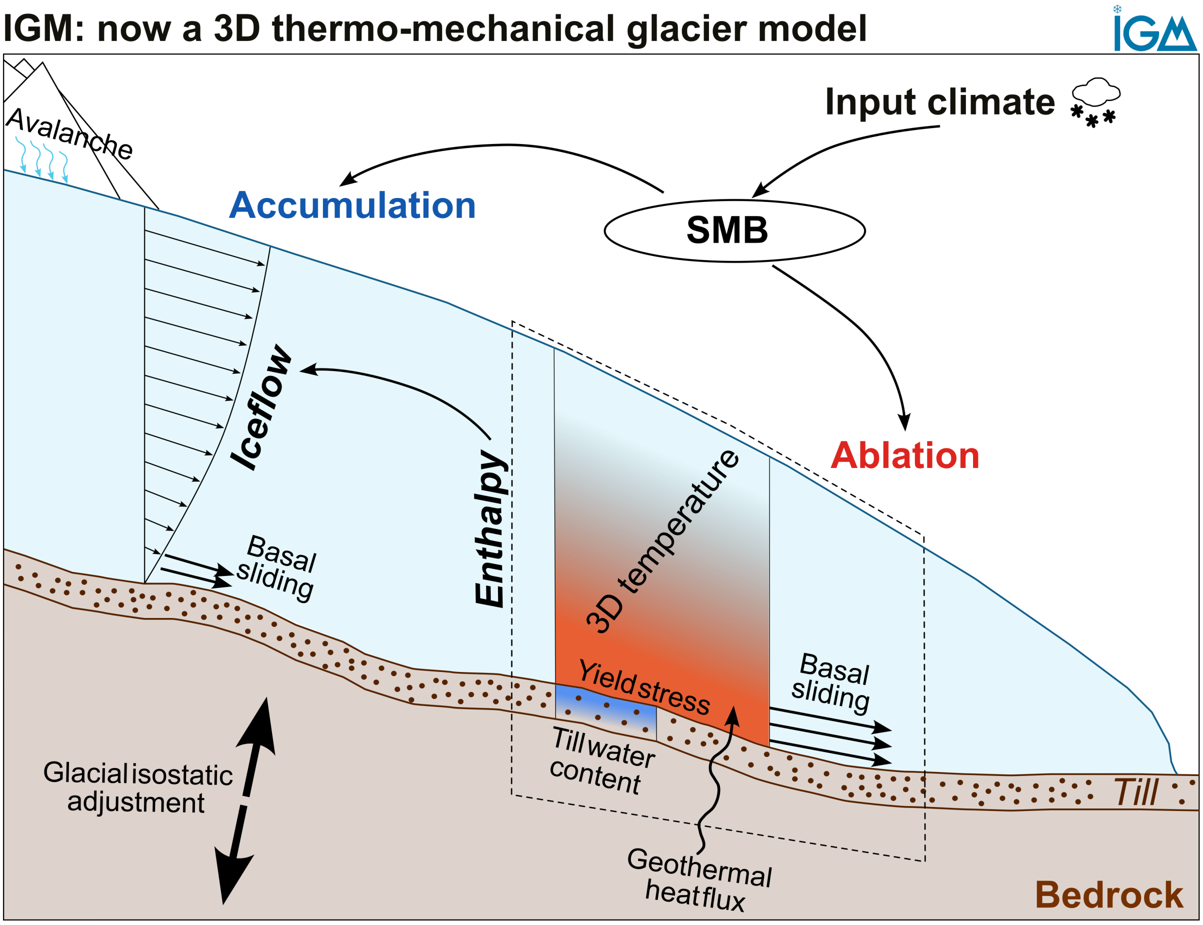

Supplementary Figure 1: Diagram showing all components of the glacier system here modelled with IGM at 300 m resolution and over the entire European Alps. “SMB” stands for “surface mass balance”.

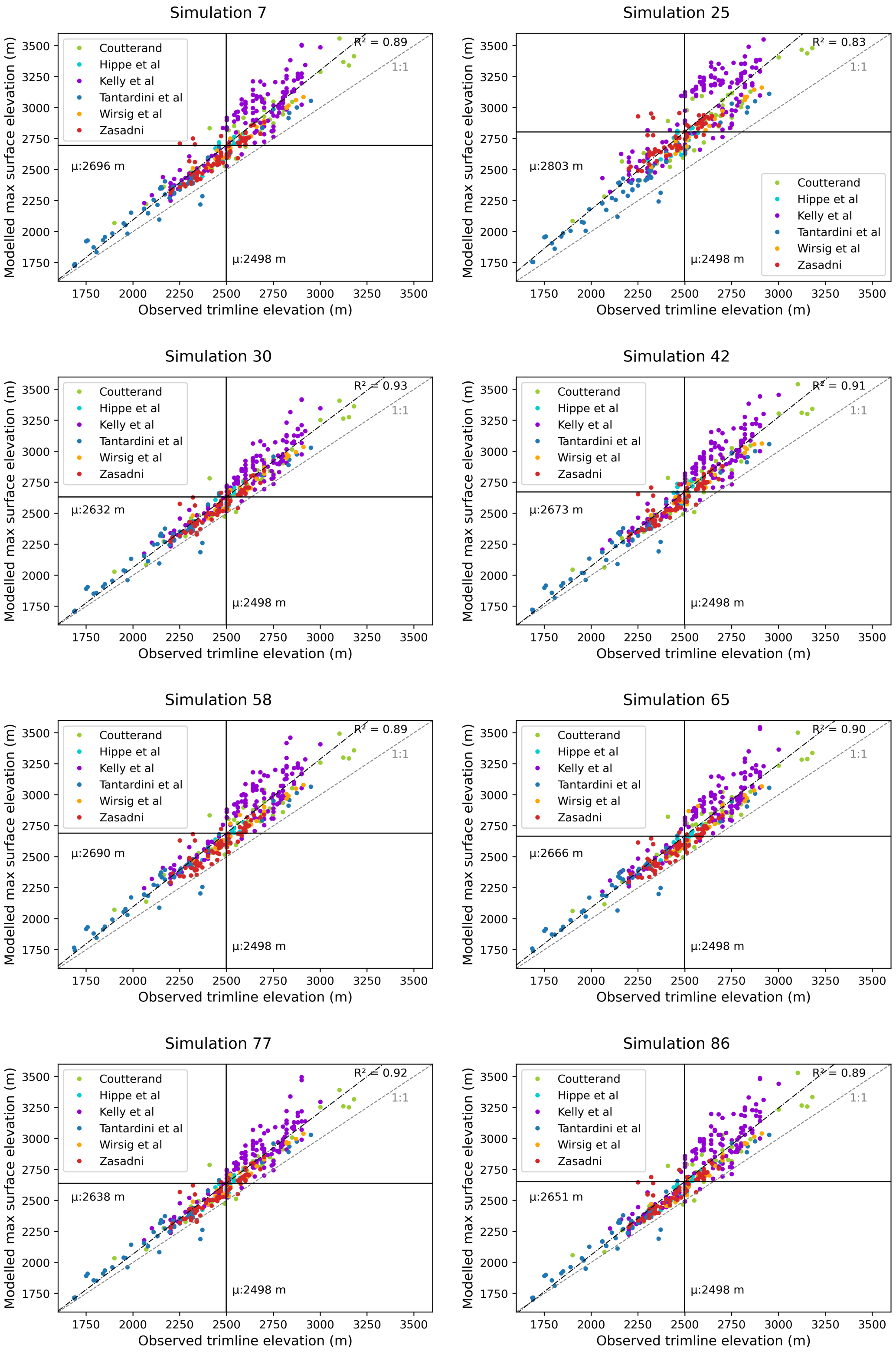

Supplementary Figure 2: Observed trimline elevations (n=353) vs time-independent maximum modelled ice surface elevations for eight randomly picked ensemble simulations (non-NROYs). Despite not targeting best-fit simulations, and the highly variable input parameter configurations between these simulations, linear regression analyses still yield a strong correlation (r^2>0.75) in all cases.

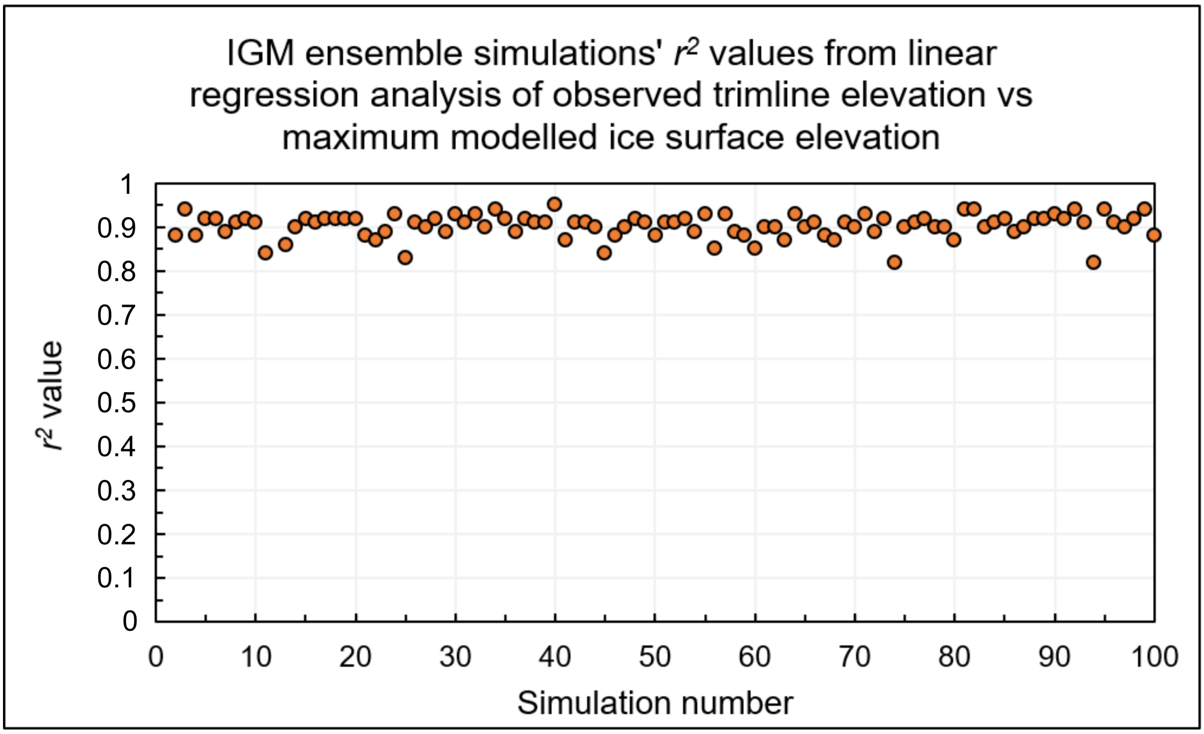

Supplementary Figure 3: R^2 values from linear regression analyses of all (n=353) observed trimline elevations compiled here versus maximum LGM modelled ice surface elevations, for all 100 ensemble simulations of the AIF ran at 300 m spatial resolution with IGM. For all simulations, and despite highly variable parameter configurations and LGM modelled AIF geometries, the linear relationship between observed and modelled elevations at the location of trimlines remains greater than 0.8, suggesting a strong correlation persists.

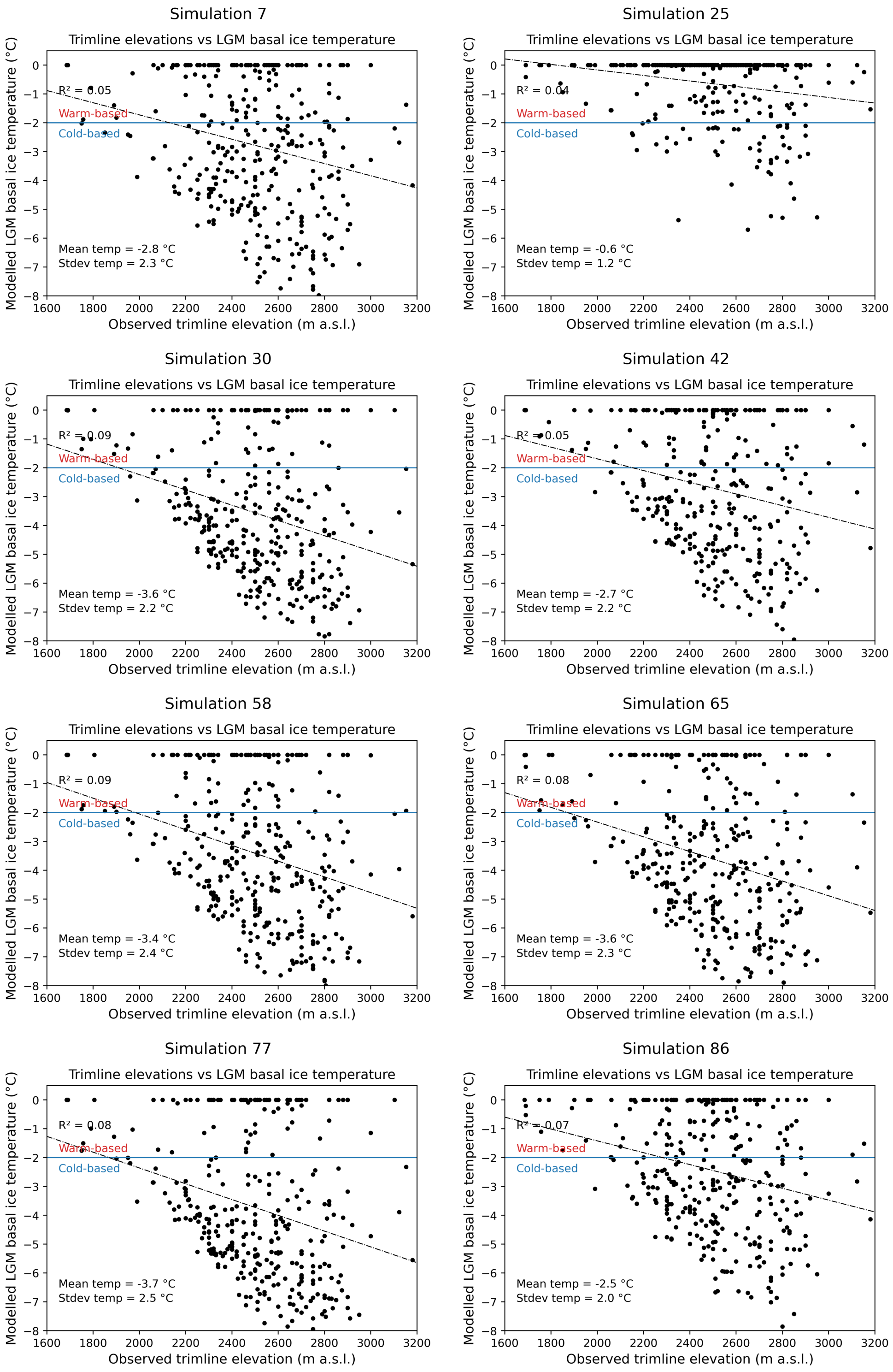

Supplementary Figure 4: Observed trimline elevations (n=353) versus maximum pressure-adjusted basal ice temperature during the LGM (26-23 ka) for eight randomly picked ensemble simulations (non-NROYs). The lack of clustering along the Y axis indicates highly variable modelled basal ice temperatures at the location of trimlines. In these graphs, a correlation between basal ice temperatures transitioning from warm to cold based and the formation of observed trimlines would produce a horizontal cluster towards a temperature value of -2 °C (blue line). This does not seem to be the case. Note that each point yields some uncertainty associated with comparing point data (observed trimlines) to a temperature value covering a 0.09 km^2 model grid cell at that location, which, over steep terrain, can introduce a certain elevation bias.

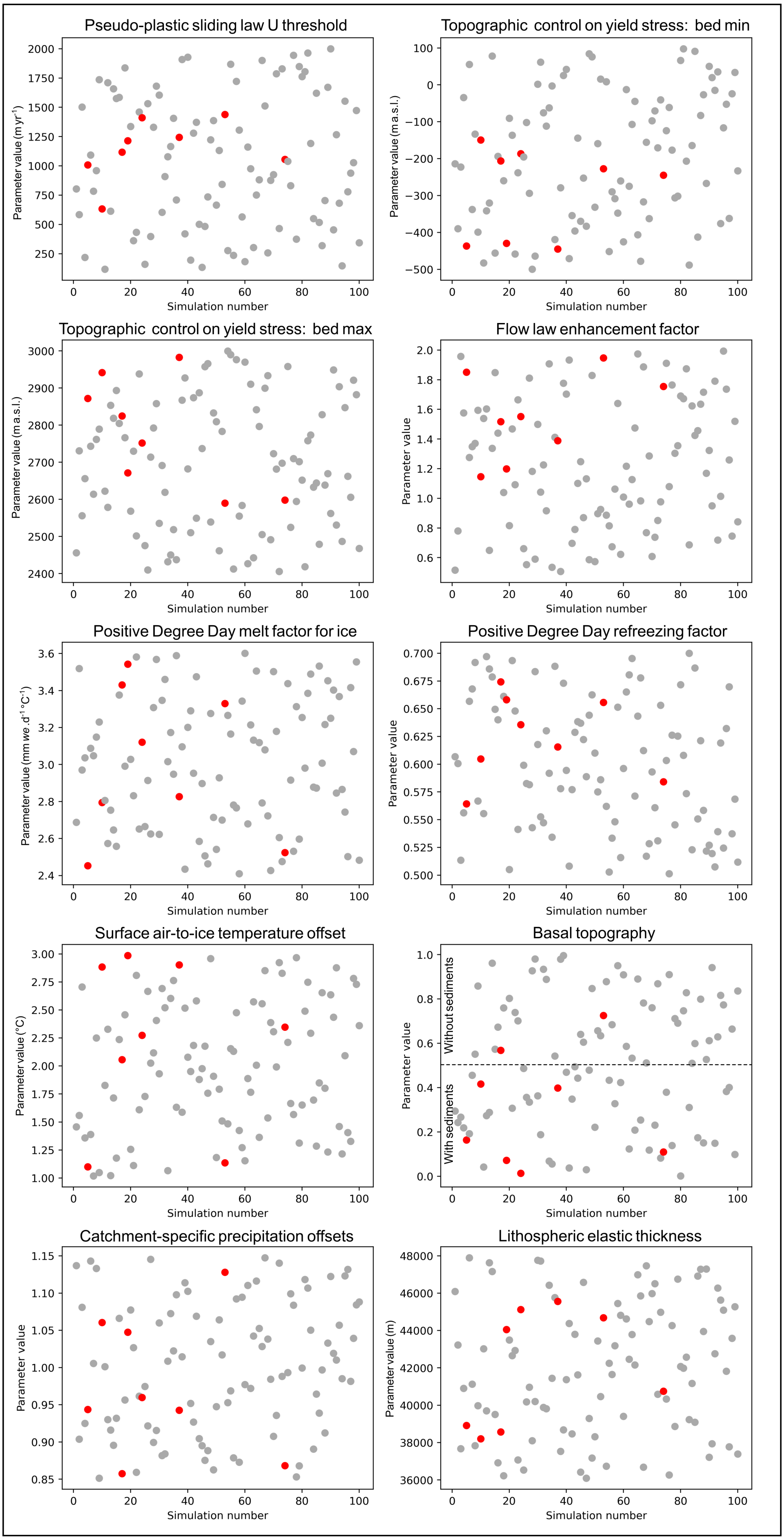
Pseudo-plastic sliding law U threshold
Parameter value (m yr⁻¹)
Topographic control on yield stress: bed min
Parameter value (m a.s.l.)
Topographic control on yield stress: bed max
Parameter value (m a.s.l.)
Flow law enhancement factor
Parameter value
Positive Degree Day melt factor for ice
Parameter value (mm we.d⁻¹ °C⁻¹)
Positive Degree Day refreezing factor
Parameter value
Surface air-to-ice temperature offset
Parameter value (°C)
Basal topography
Without sediments
With sediments
Parameter value
Catchment-specific precipitation offsets
Parameter value
Lithospheric elastic thickness
Parameter value (m)
Simulation number

Supplementary Figure 5: Ensemble-varying parameter values for the NROY (red, *n*=8) and all remaining (grey, *n*=92) ensemble simulations, sampled using a Latin Hypercube algorithm. The wide ranges in NROY parameter values (red) along the Y axes indicate a lack of clusters. This suggests better model-data fit (relative to other ensemble simulations) can be obtained with highly variable individual parameter values, making the variety of suitable parameter configurations difficult to predict without a comprehensive exploration of the parameter space. These results indicate the relationship between model-data fit and parameter values may feature numerous local minima. This justifies the use of a perturbed parameter ensemble approach for exploring the numerous possible model responses to parameter variations.

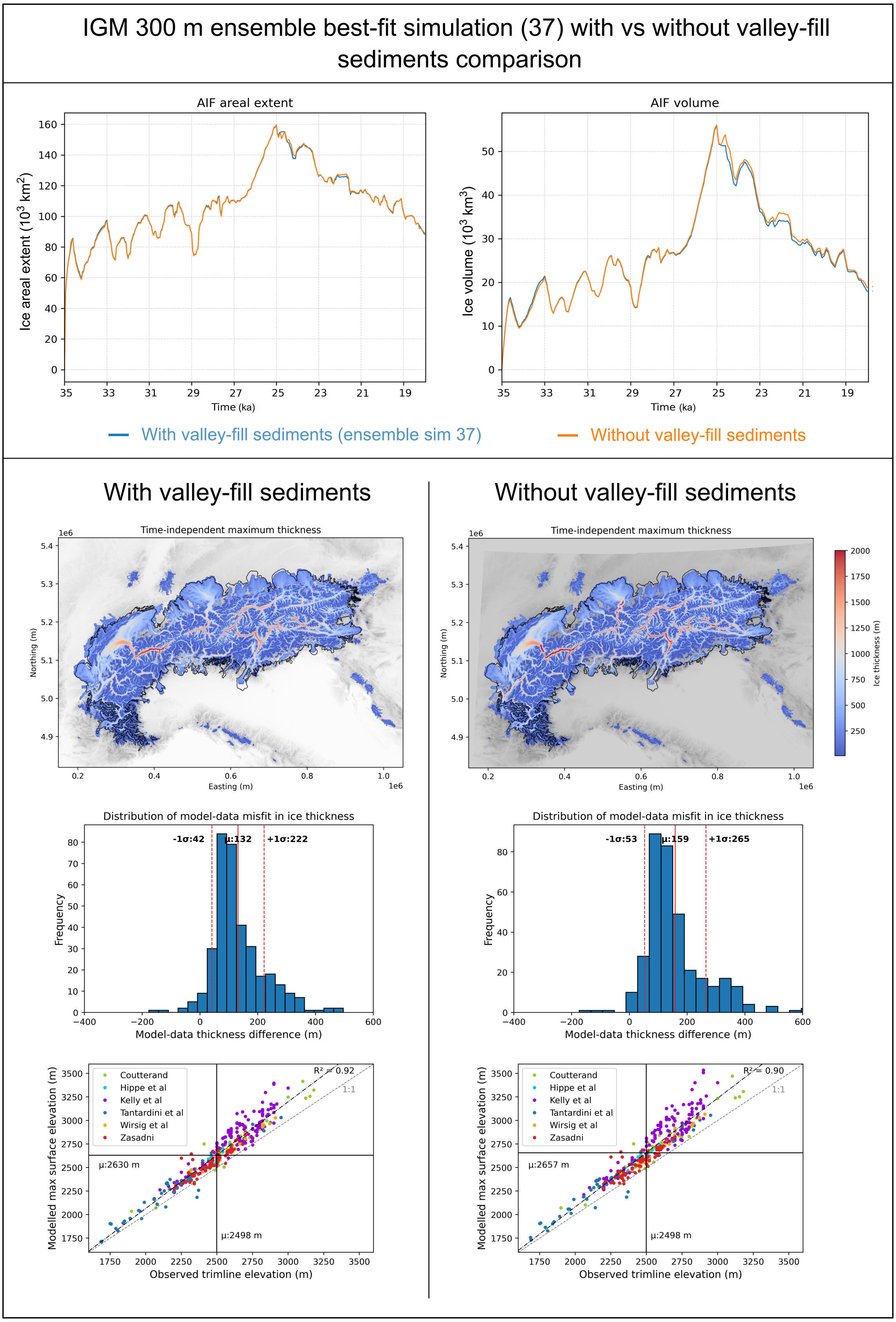

Supplementary Figure 6: Sensitivity of ensemble best-fit simulation (37) to removal of valley-fill sediments tested by running IGM with the original DEM (with sediments) and with the topography from Mey *et al*. (2016) without valley-fill sediments. This test shows that the impact of removing valley-fill sediment on the modelled LGM geometry of the AIF is minimal.

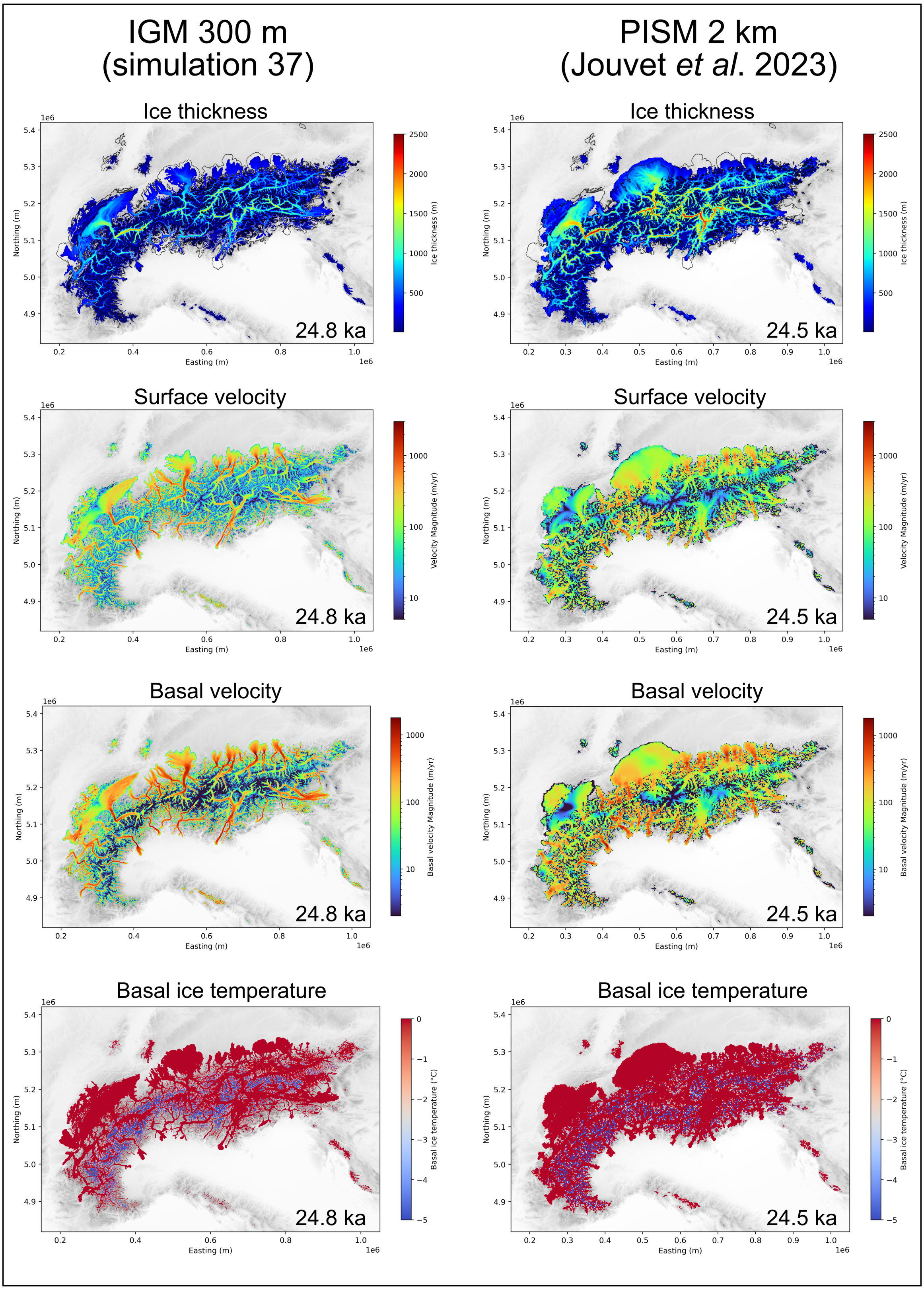

Supplementary Figure 7: Qualitative comparisons of spatially dependent output variables towards the LGM (24.8 ka) from ensemble simulation 37, *i.e*. our best-fitting IGM 300 m resolution simulation, and Jouvet *et al*. (2023)' PISM 2 km simulation.

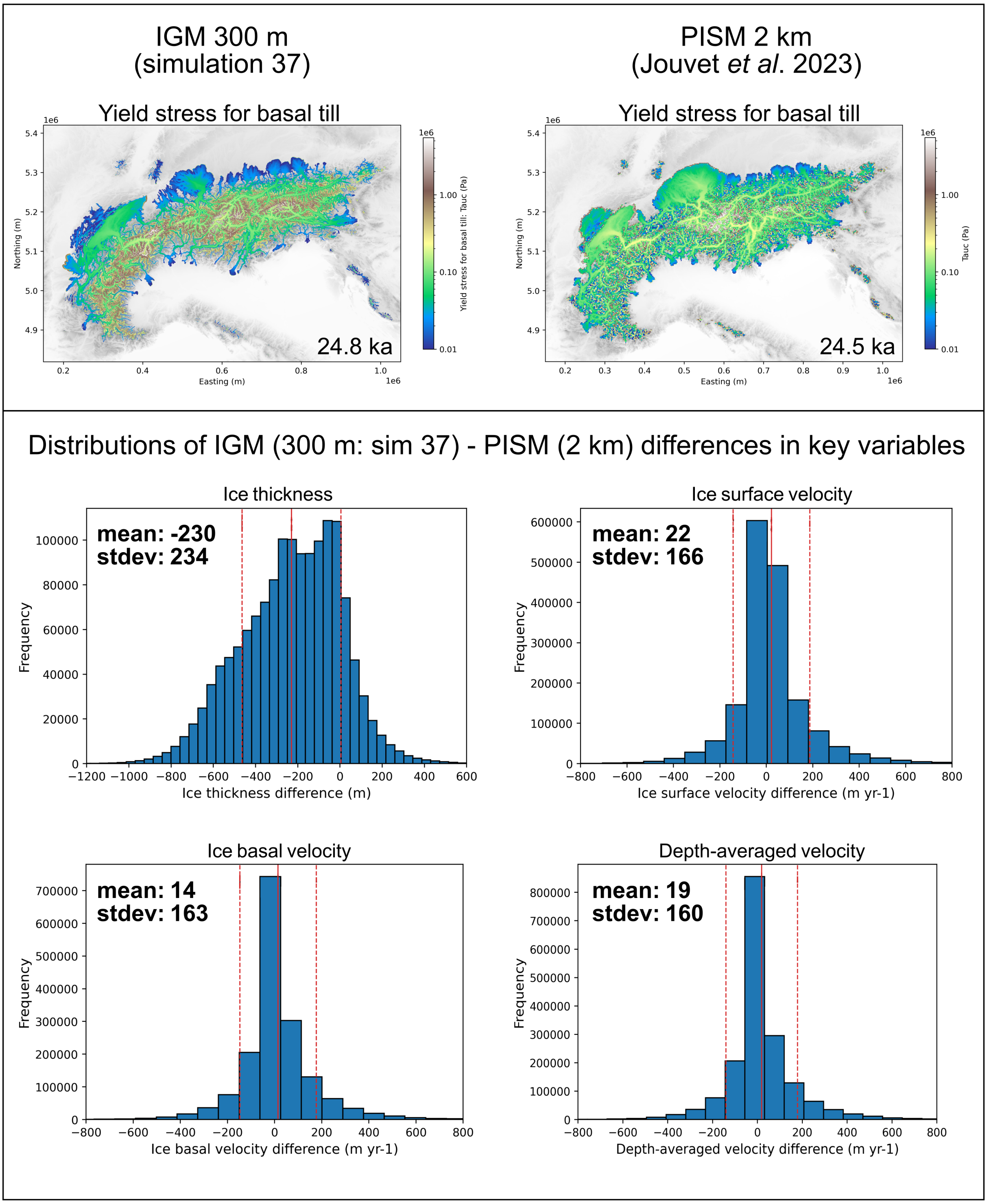

Supplementary Figure 8: Qualitative and quantitative comparisons of spatially-dependent output variables towards the LGM (24.8 ka) from ensemble simulation 37, *i.e.* our best-fitting IGM 300 m simulation, and Jouvet *et al.* (2023)' PISM 2 km simulation.

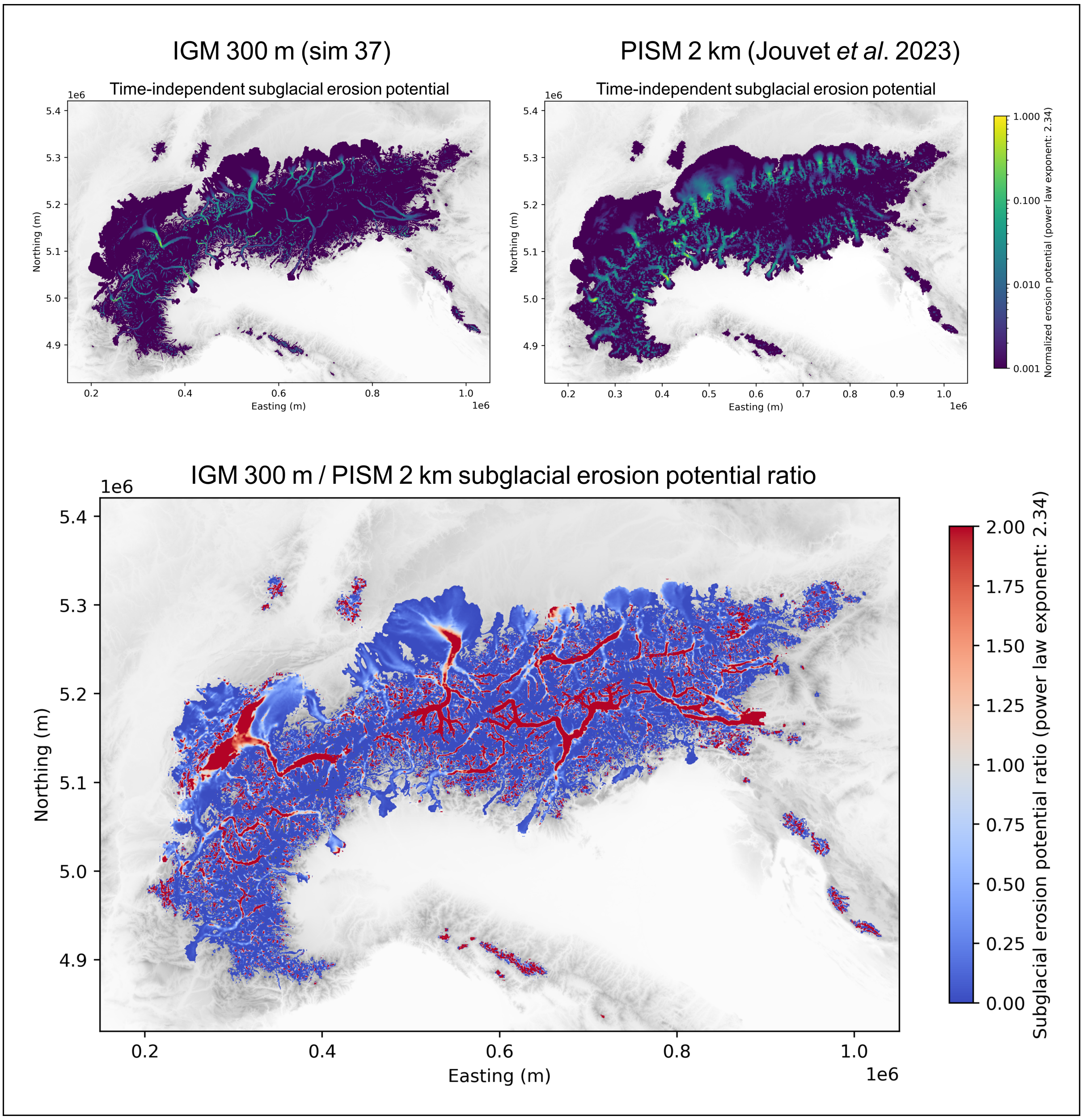

Supplementary Figure 9: Time-integrated subglacial erosion potential for IGM 300 m best-fitting simulation 37 (upper left panel), for the PISM 2 km simulation of Jouvet *et al.* (2023) (upper right panel), and the ratio between these two (lower panel). The subglacial erosion potential is here computed (for both models) using a simple power law and a velocity exponent of 2.34, after Koppes *et al.* (2015) and Seguinot *et al.* (2021). Red colours indicate more subglacial erosion potential with IGM 300 m than with PISM at 2 km, blue colours indicate the opposite. At higher resolution, modelled basal ice velocities become significantly faster mostly in main valley troughs.

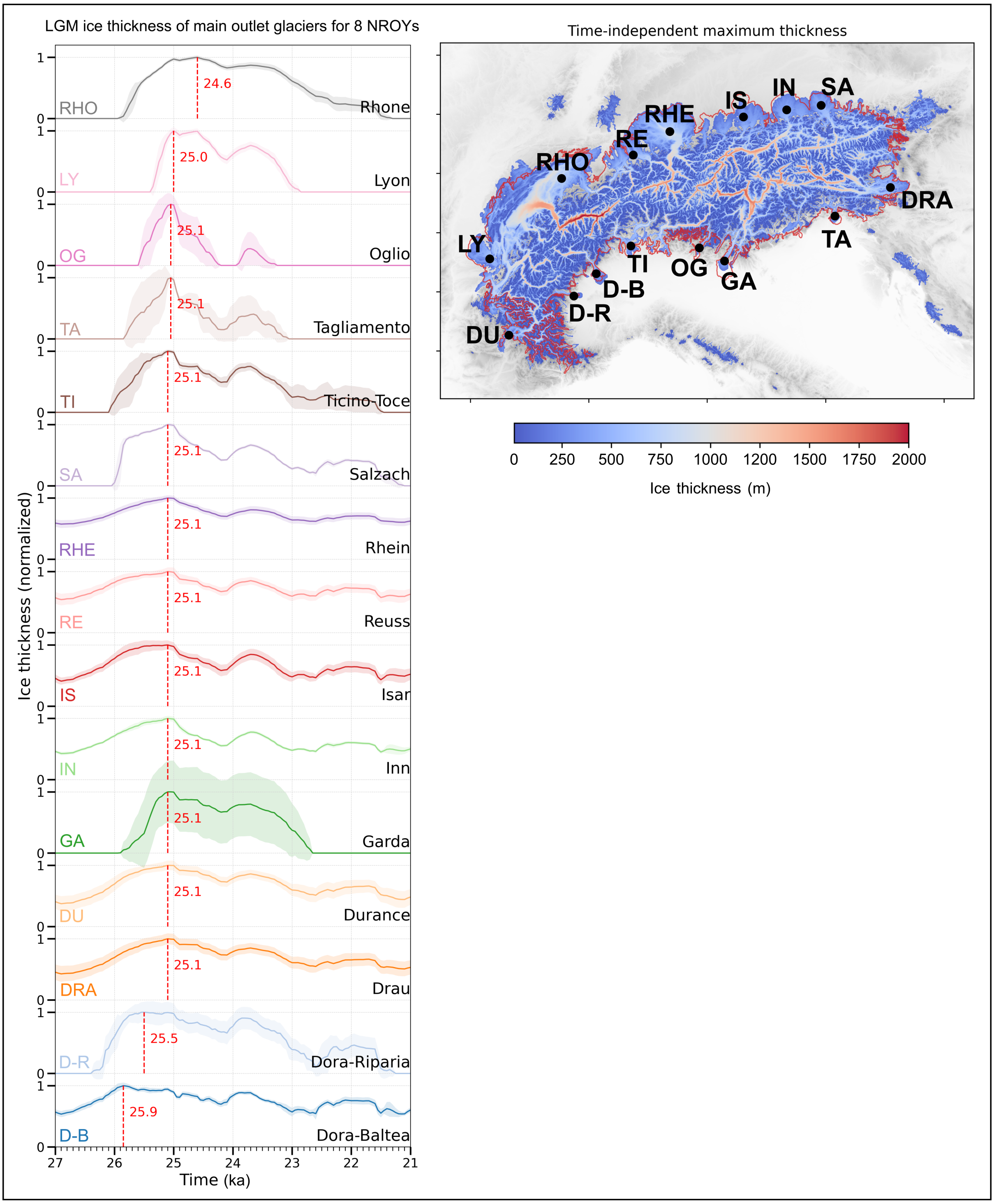

Supplementary Figure 10: Normalized ice thickness time series over the full LGM period at the location of black dots highlighted in right-hand map. The location of dots is chosen such that extracted ice thickness time series act as a proxy for the ice extent evolution of the 15 largest AIF outlet glaciers during the LGM. Thick lines and transparent bands indicate the NROY mean (n=8) and standard deviation of the ice thickness data, respectively. Dashed red lines indicate the timing of maximum outlet glacier thickness and thus extent, in ka. These time series suggest centennial-scale asynchronies in the timing of LGM outlet glacier extent. For instance, the Lyon and Rhone glacier tongues reach (or remain close to) their maximum extents until five centuries after most other glaciers start to retreat. On the other hand, the Dora-Baltea and Dora-Riparia glaciers reach maximum extents eight and four centuries before other sampled glaciers.

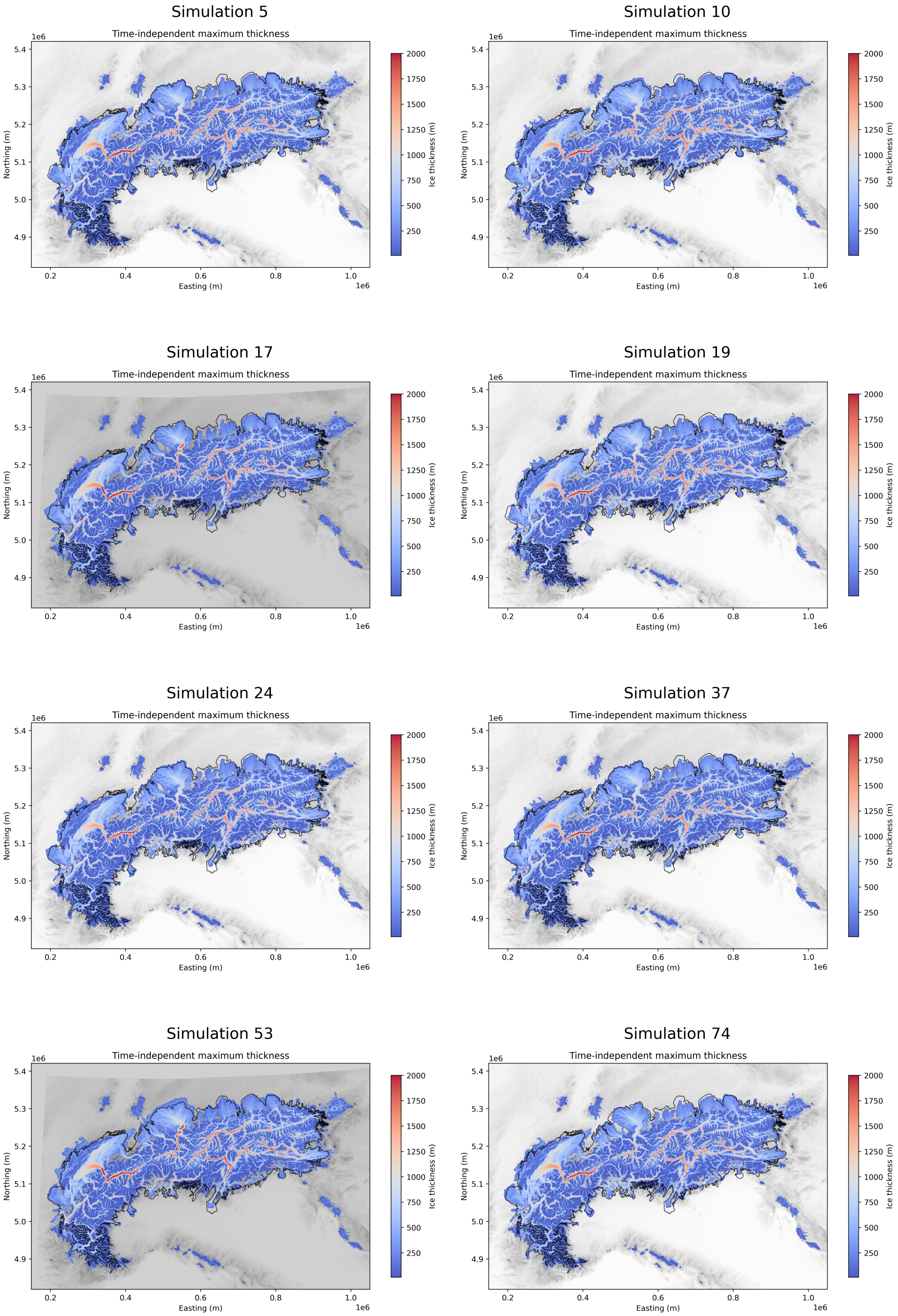

Supplementary Figure 11: Time-independent maximum ice thickness for the eight NROY ensemble-member simulations that remain after applying three model-data comparison sieves.

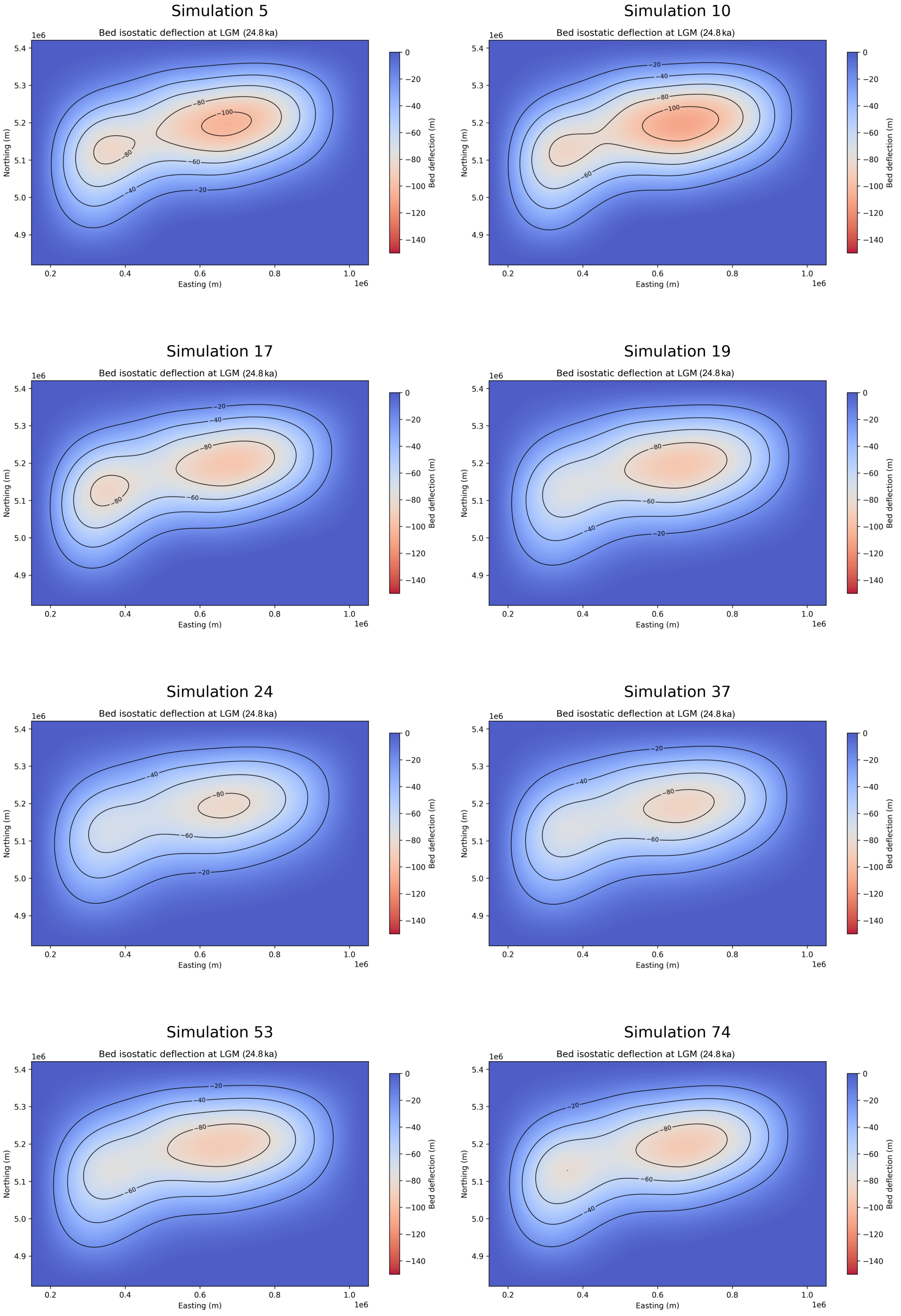

Supplementary Figure 12: Glacial Isostatic Adjustment-induced crustal deflection at the LGM for the eight NROY ensemble-member simulations that remain after applying three model-data comparison sieves. During the LGM, we find our NROYs produce a maximum crustal deflection near the AIF centre of 93.1 ± 13.5 m (NROY mean ± stdev).

Supplementary Table 2: Values and descriptions for all non-ensemble-varying IGM parameters

Supplementary Table 2. non-varying IGM parameter values

IGM parameter name	Description	System component	Value (fixed for all ensemble simulations)	Unit
clim_update_freq	Frequency at which the climate is updated using glacial index	Input climate	100.0	yr
smb_accpdd_update_freq	Update the Surface Mass Balance (SMB) each x years	Surface mass balance	1.0	yr
smb_accpdd_thr_temp_snow	Threshold temperature for solid precipitation	Surface mass balance	0.0	°C
smb_accpdd_thr_temp_rain	Threshold temperature for liquid precipitation	Surface mass balance	2.0	°C
smb_accpdd_melt_factor_snow	Positive Degree Day melt rate for snow	Surface mass balance	1.20409532638	m $°C^{-1}$ yr^{-1}
smb_accpdd_shift_hydro_year	This serves to start Oct 1. the acc/melt computation	Surface mass balance	0.75	yr
smb_accpdd_ice_density	Density of ice for conversion of SMB into ice equivalent	Surface mass balance	910.0	kg m^{-3}
smb_accpdd_wat_density	Density of water	Surface mass balance	1000.0	kg m^{-3}
iflo_regu_weertman	Regularization parameter for Weertman's sliding law	Basal sliding	10^{-10}	n/a
iflo_exp_glen	Glen's flow law exponent	Ice flow	3.0	n/a
iflo_exp_weertman	Weertman's law exponent	Basal sliding	4.0	n/a
iflo_gravity_cst	Acceleration due to gravity of a free falling object	Ice flow	9.81	m s^{-2}
iflo_ice_density	Density of ice	Ice flow	910.0	kg m^{-3}
iflo_Nz	Number of grid points for the vertical discretization	Ice flow	10.0	n/a
iflo_vert_spacing	Discretization density to get more points towards bed than surface	Ice flow	4.0	n/a
iflo_thr_ice_thk	Threshold ice thickness for computing strain rate	Ice flow	0.1	m
iflo_dim_arrhenius	Dimension of the arrhenius factor (horizontal 2D or 3D)	Ice flow	3.0	n/a
iflo_retrain_emulator_freq	Frequency at which the emulator is retrained, 0 means never	Neural network	7.0	time steps
iflo_retrain_emulator_lr	Learning rate for the retraining of the emulator	Neural network	10^{-5}	n/a
iflo_retrain_emulator_nbit	Number of iterations at each time step for retraining the emulator	Neural network	1.0	iterations
iflo_force_max_velbar	Artifically upper-bound of ice velocities	Ice flow	3000.0	m yr^{-1}
iflo_network	The type of network, it can be cnn or unet	Neural network	"cnn"	n/a
iflo_nb_layers	Number of layers in the Convolutional Neural Network (CNN)	Neural network	16.0	n/a
iflo_nb_out_filter	Number of output filters in the CNN	Neural network	32.0	n/a
iflo_conv_ker_size	Size of the convolution kernel	Neural network	3.0	n/a
iflo_min_sr	Minimum strain rate	Ice flow	10^{-5}	yr^{-1}
iflo_max_sr	Maximum strain rate	Ice flow	1.0	yr^{-1}
time_start	Simulation start	Time	-35000.0	yr BP
time_end	Simulation end	Time	-18000.0	yr BP
time_save	Save output variable frequency	Time	50.0	yr
time_cfl	CFL number for the stability of the mass conservation scheme	Time	0.3	…..
time_step_max	Maximum time step allowed, used only with slow ice	Time	10.0	yr
thk_slope_type	Slope limiter for the ice thickness equation (godunov or superbee)	Ice flow	"superbee"	n/a
vflo_method	Method to retrieve vertical velocities (kinematic, incompressibility)	Ice flow	"incompressibility"	n/a
enth_water_density	Density of water	Enthalpy	1000.0	kg m^{-3}
enth_spy	Number of seconds in a year	Enthalpy	31556926.0	seconds yr^{-1}
enth_ki	Conductivity of cold ice	Enthalpy	2.1	W m^{-1} K^{-1}
enth_ci	Specific heat capacity of ice	Enthalpy	2009.0	W s kg^{-1} K^{-1}
enth_Lh	Latent heat of fusion	Enthalpy	334000.0	W s kg^{-1}
enth_KtdivKc	Ratio of temperate versus cold ice diffusivity	Enthalpy	0.1	n/a
enth_claus_clape	Clausius-Clapeyron constant	Enthalpy	7.9 x 10^{-8}	K Pa^{-1}
enth_melt_temp	Melting point at standart pressure	Enthalpy	273.15	K
enth_ref_temp	Reference temperature	Enthalpy	223.15	K
enth_till_friction_angle_phi_min	Minimum till friction angle in bed-elevation dependent scheme	Yield stress	15.0	°
enth_till_friction_angle_phi_max	Maximum till friction angle in bed-elevation dependent scheme	Yield stress	50.0	°
enth_drain_rate	Water draining rate	Yield stress	0.001	mm yr^{-1}
enth_till_wat_max	Maximum water till tickness	Yield stress	2.0	m
enth_tauc_min	Lower caping bound for yield stress	Yield stress	10000.0	Pa
enth_tauc_max	Upper caping bound for yield stress	Yield stress	10000000000.0	Pa
avalanche_update_freq	Update frequency of the avalanche module	Avalanche	5.0	yr
avalanche_angleOfRepose	Angle of repose. For bed slopes above this, ice "avalanches"	Avalanche	45.0	°
gflex_update_freq	Update frequency of the gFlex GIA module	Glacial isostatic adjustment	50.0	yr
gflex_dx	Spatial grid resolution of the gFlex GIA module	Glacial isostatic adjustment	2000.0	m

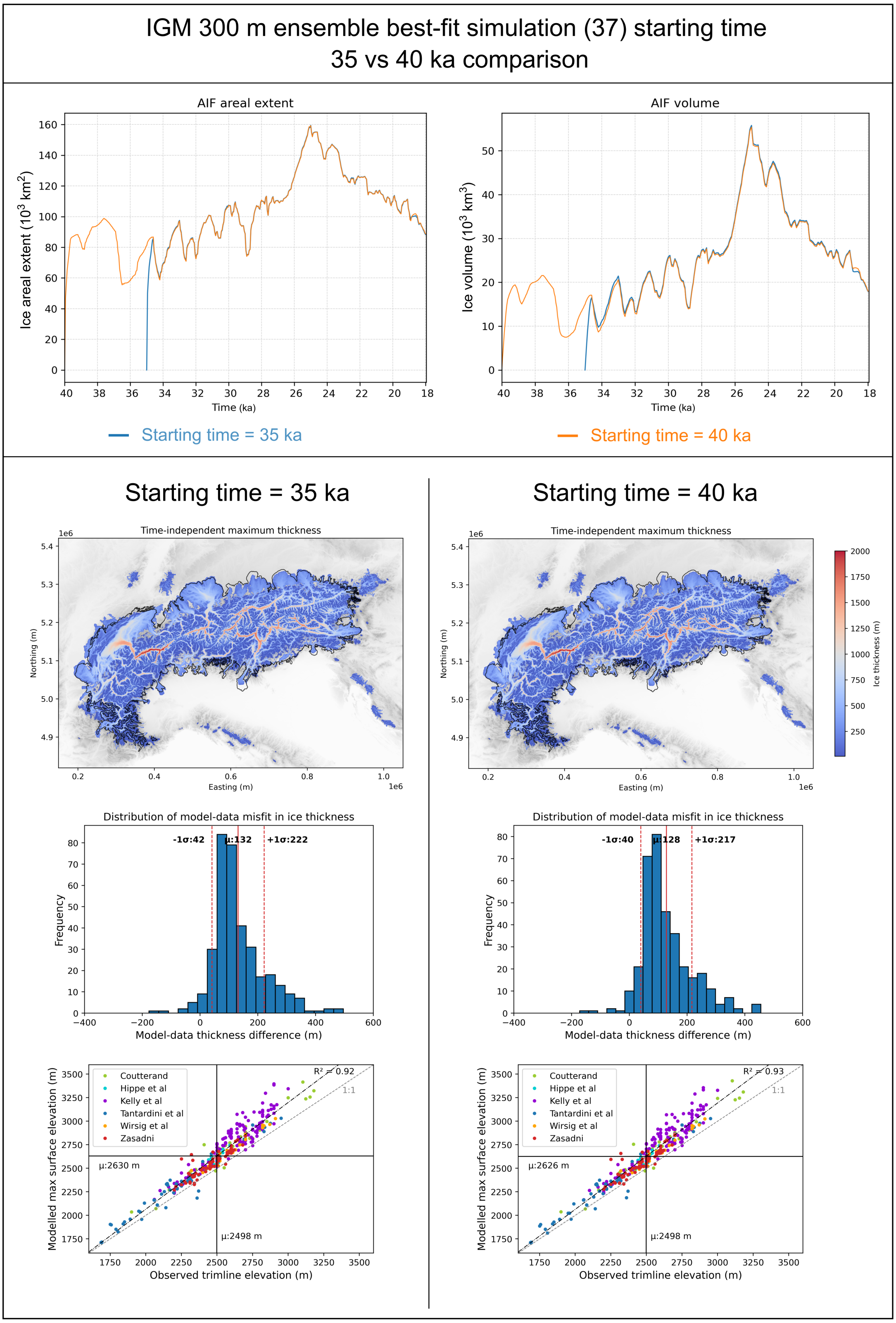

Supplementary Figure 13: Sensitivity of ensemble best-fit simulation (37) to starting time tested by running two simulations starting at 40 ka and 35 ka (the starting time of our ensemble simulations), respectively. This test shows the two model states converge within 4 kyr of simulation, leading to nearly identical results at the LGM. Although unrealistic, starting our simulations from an ice-free topography at 35 ka does not lead to biases in the LGM state (10 kyr later) of the AIF, due to the ice field memory and inertia not exceeding 4-6 kyr.

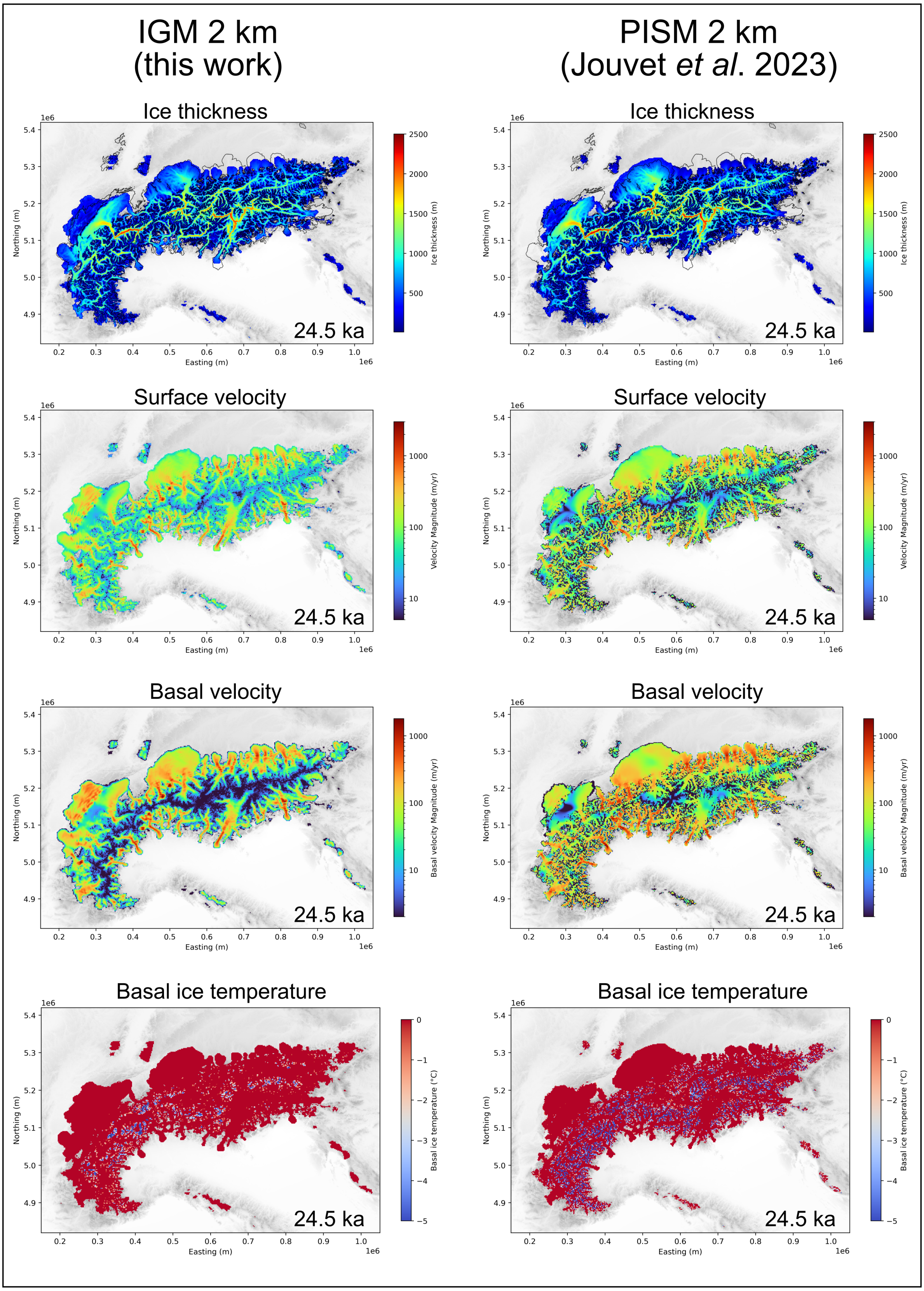

Supplementary Figure 14: Qualitative comparisons of spatially dependent output variables at the modelled LGM in the European Alps (24.5 ka) from an IGM 2 km simulation (see 'Methods' section 'Model validation' in main paper) and Jouvet *et al*. (2023)' 2 km PISM simulation, using a similar model setup. This comparison exercise was conducted to validate the use of IGM for Alpine Ice Field-wide LGM simulations.

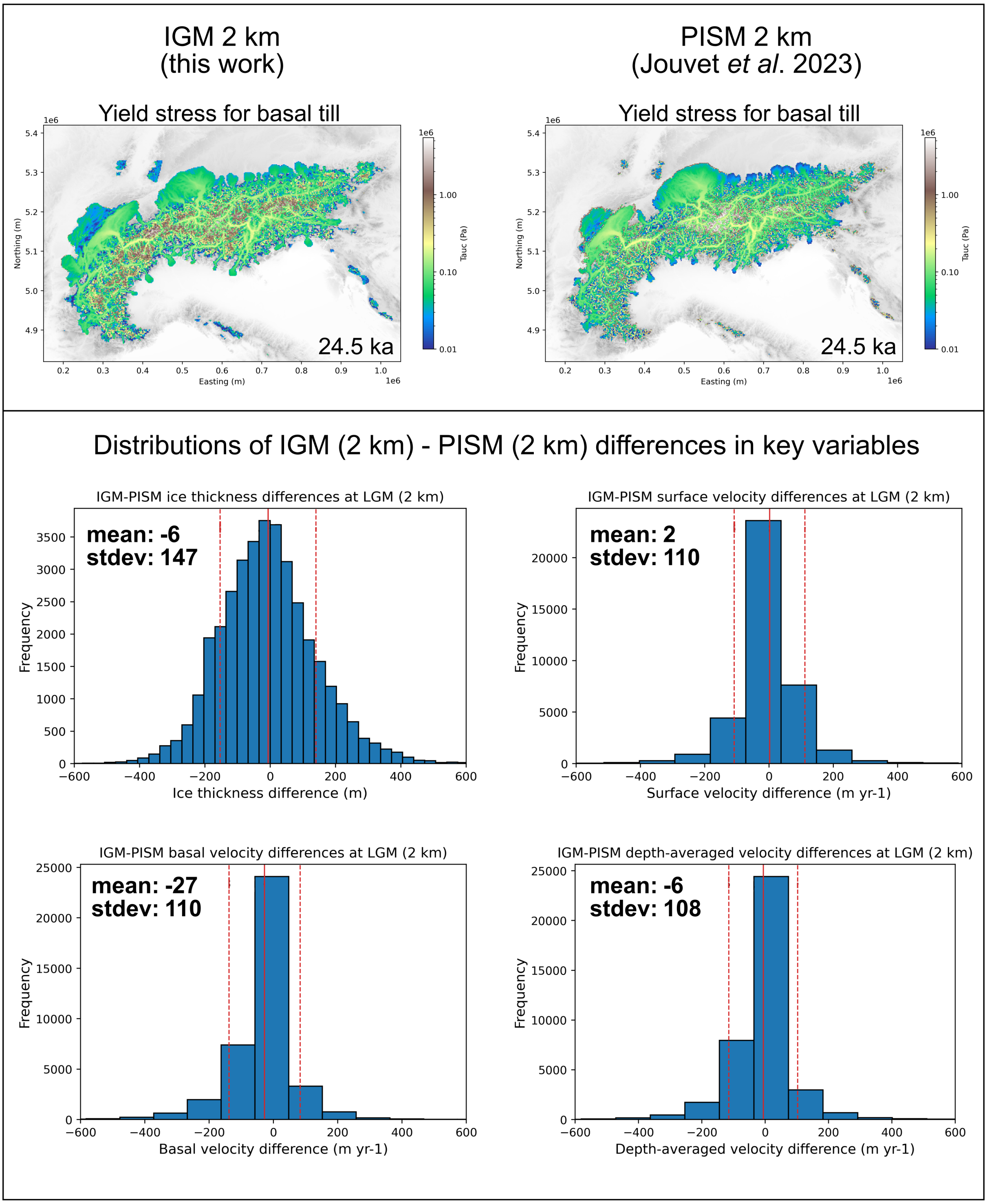

Supplementary Figure 15: Qualitative and quantitative comparisons of spatially dependent output variables at the modelled LGM (with EPICA glacial index forcing: 24.5 ka) between an IGM 2 km simulation (see 'Methods' section in main paper) and Jouvet *et al*. (2023)' PISM 2 km simulation, using a similar model setup. This comparison exercise was conducted to validate the use of IGM for Alpine Ice Field-wide LGM simulations.

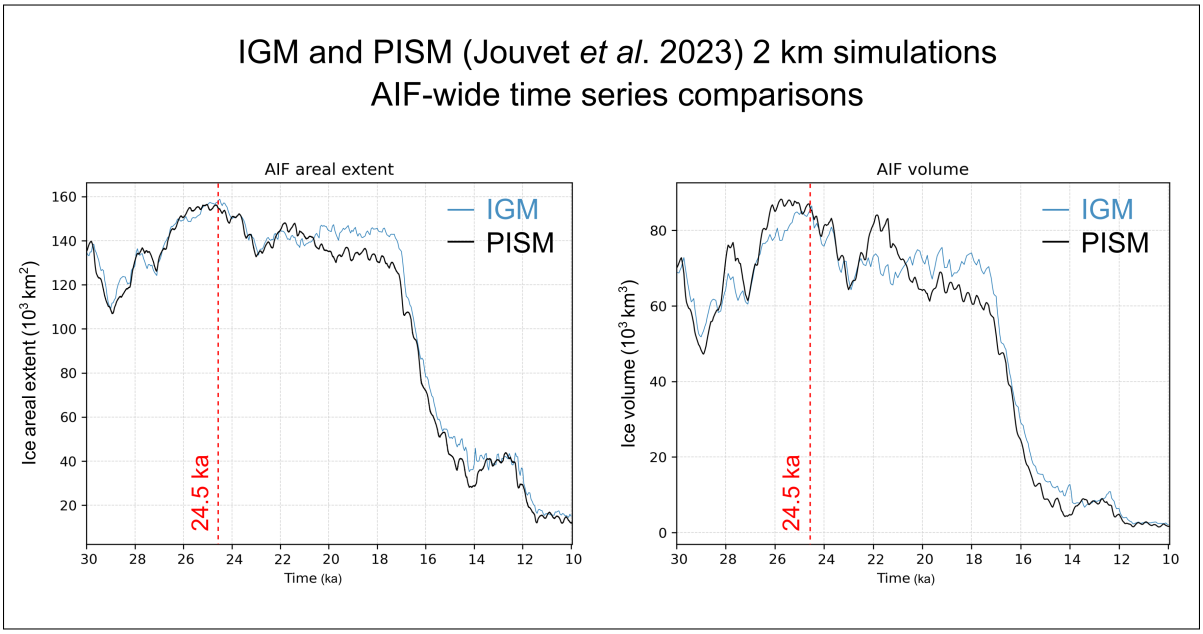

Supplementary Figure 16: Time series of the Alpine Ice Field areal extent and volume evolution between 30 and 10 ka for both IGM and PISM (Jouvet *et al*. 2023) simulations at 2 km, under a comparable model setup. This comparison exercise was conducted to validate the use of IGM for AIF-wide LGM simulations.

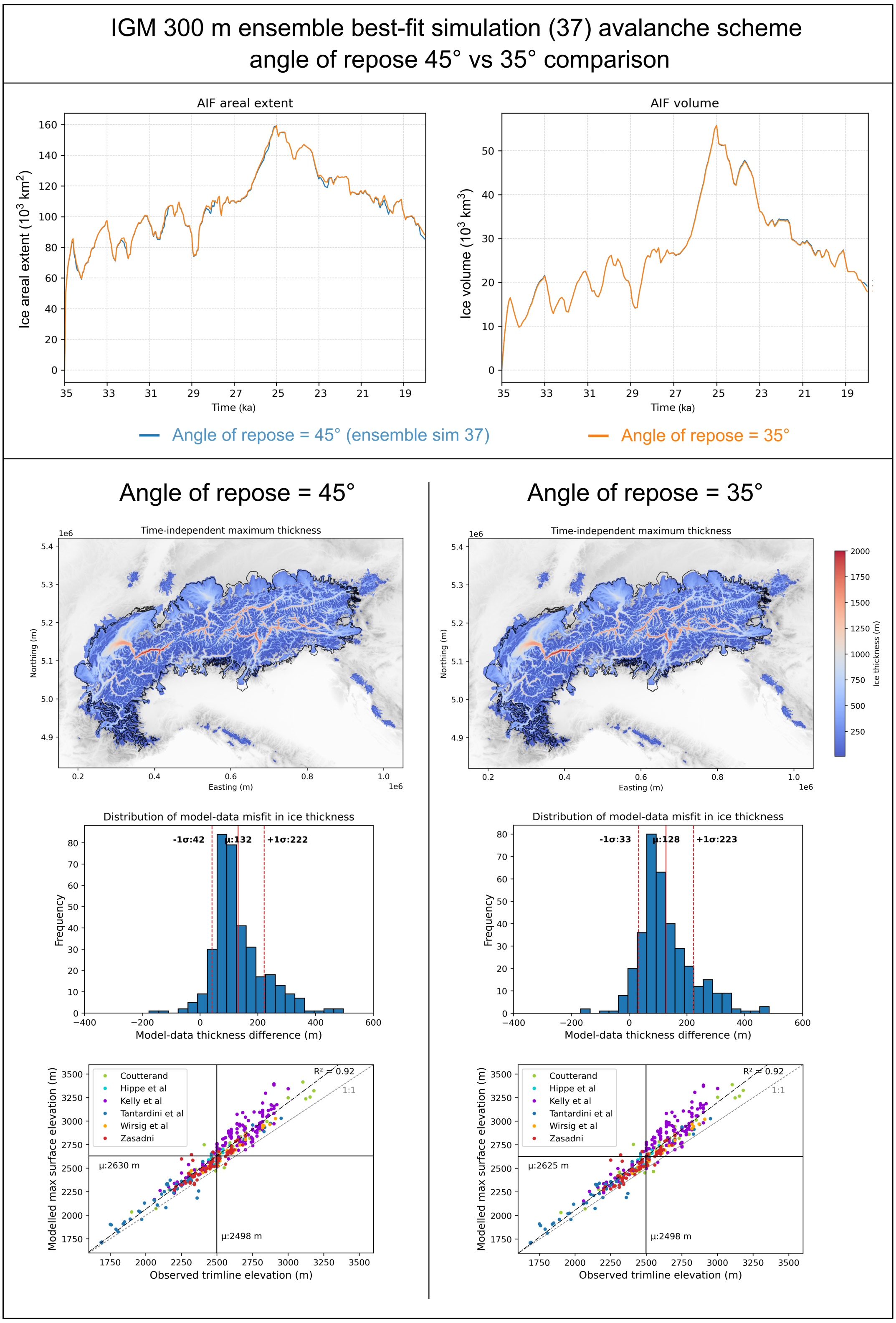

Supplementary Figure 17: Sensitivity of ensemble best-fit simulation (37) avalanche scheme angle of repose parameter, tested by running two simulations starting with values of 35° and 45° (the value for ensemble simulation 37), respectively. These values bracket the range of typical values for glacier angle of reposes. This test shows little difference in LGM model-data fit between the two simulations, highlighting a lack of model sensitivity to the angle of repose parameter value, when analysing Alps-wide LGM thickness and extent fit.

Supplementary Table 3: List and descriptions of high-resolution Digital Elevation Models used for independent verification of reported trimline elevations.

Supplementary Table 3. Digital elevation models used for independent verification of trimline elevations

DEM name	Region covered	Releasing organisation	Year released	Spatial resolution (m)	Vertical error (m)	URL
RGE ALTI® Version 2.0 product	French Alps	Institut national de l'information géographique et forestière	2024	1.0	0.2 - 10.0	https://geoservices.ign.fr/documentation/donnees/alti/rgealti
swissALTI3D	Swiss Alps	Federal Office of Topography swisstopo	2024	0.5	0.3 - 1.0	https://www.swisstopo.admin.ch/en/height-model-swissalti3d
DigitalTerrainModel-0.5m	Italian Alps, south Tyrol region	Autonome Provinz Bozen - Abteilung Natur, Landschaft und Raumentwicklung - Amt für Landesplanung und Kartografie	2021	0.5	0.2 - 10.0	https://data.civis.bz.it/de/dataset/modello-digitale-del-terreno-dtm-05m
DTM 5X5 - Modello digitale del terreno	Italian Alps, Lombardy region	Regione Lombardia	2015	5.0	0.3 - 2.0	https://www.geoportale.regione.lombardia.it/metadati?p_p_id=detailSheetMetadata_WAR_gptmetadataportlet&p_p_lifecycle=0&p_p_state=normal&p_p_mode=view&_detailSheetMetadata_WAR_gptmetadataportlet_identifier=r_lombar%3Adfc98d60-5f02-4a2b-8113-5ad24cc53a9c&_jsfBridgeRedirect=true
RIPRESA AEREA ICE 2009-2011 - DTM 5	Italian Alps, Piemonte region	Regione Piemonte	2019	5.0	0.3	https://www.geoportale.piemonte.it/geonetwork/srv/ita/catalog.search#/metadata/r_piemon:224de2ac-023e-441c-9ae0-ea493b217a8e
Modello Digitale del Terreno (DTM 2005/2008)	Italian Alps, Valle d'Aosta region	Regione Autonoma Valle d'Aosta	2008	2.0	0.2 - 10.0	https://geoportale.regione.vda.it/download/dtm/
Digitales Geländemodell Tirol product	Austrian Alps, Tyrol region	Land Tirol	2023	0.5-1.0	0.2 - 10.0	https://www.data.gv.at/katalog/dataset/land-tirol_tirolgelnde/resource/3eb16718-a4fe-45e3-a92c-3d057237ae00#additional-info

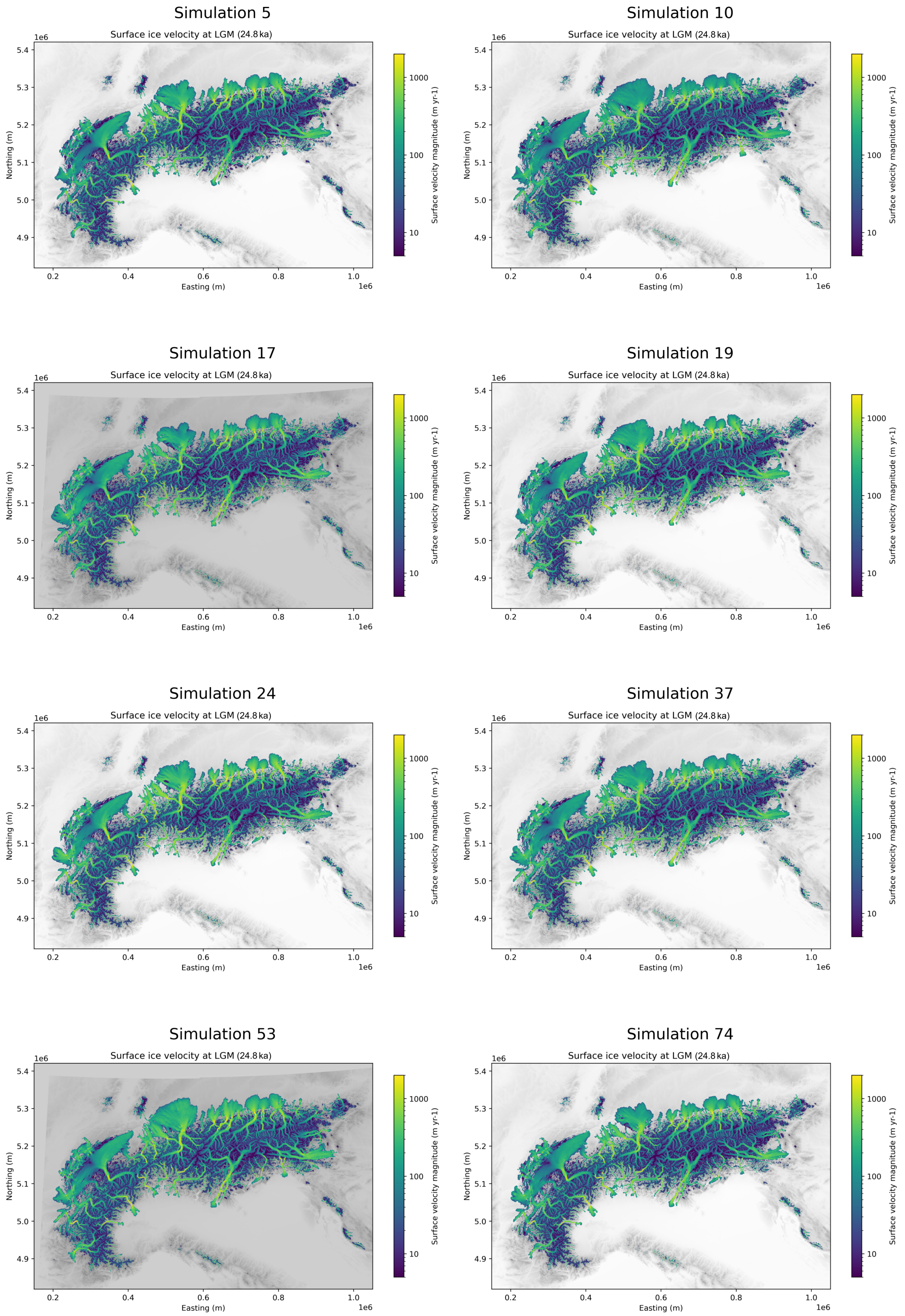

Supplementary Figure 18: LGM ice surface velocity (24.8 ka) for the eight NROY ensemble-member simulations that remain after applying three model-data comparison sieves.

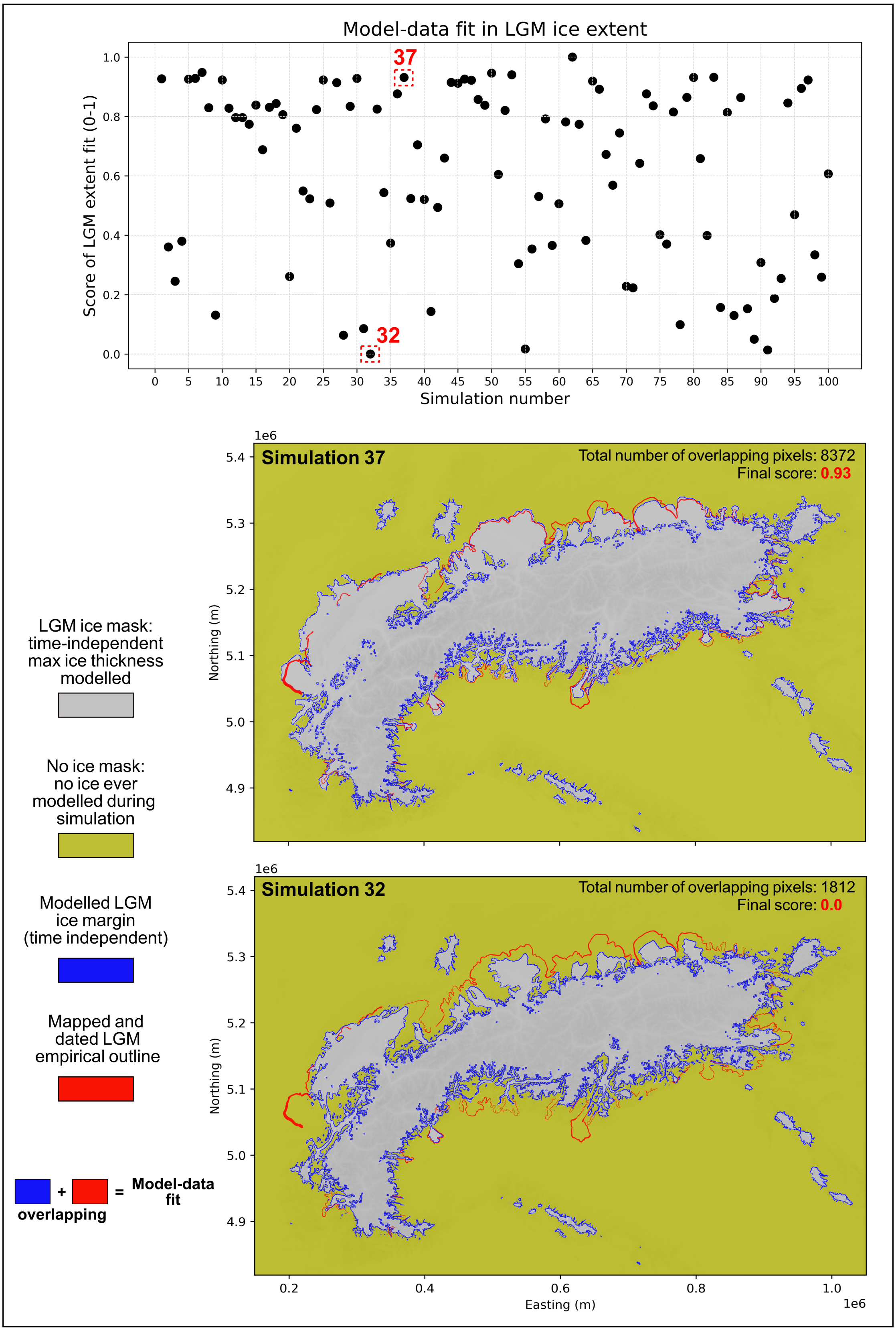

Supplementary Figure 19: Examples of quantitative and automatized scoring for LGM ice-extent fit for two ensemble simulations, *i.e.* simulation 37 (overall best-fit ensemble simulation) and simulation 32 (worse fit at the extent test). Normalized scores for all ensemble simulations are shown in the top panel. This test computes the total number of overlapping pixels between the modelled LGM ice margin (blue), and the empirical LGM outline (red) filtered to only feature

regions of good confidence in LGM margin due to abundant mapping and dating. The modelled LGM ice margin is obtained automatically by dilating pixels from the ice-free mask (time-independent, olive green colour in the two bottom panels) towards pixels of the ice mask (time-independent, light grey colour in the two bottom panels). For the dilation size, we use a kernel of 7x7 pixels. This test is used as Sieve 1 in our model-data comparison analysis, using a minimum threshold value of 0.8 (see Figure 2 in main paper). Any simulation scoring above 0.8 are retained and passed on to sieve 2 and 3, which then assess the mean and stdev of the misfit between maximum modelled ice surface elevations and reported trimline elevations, respectively.

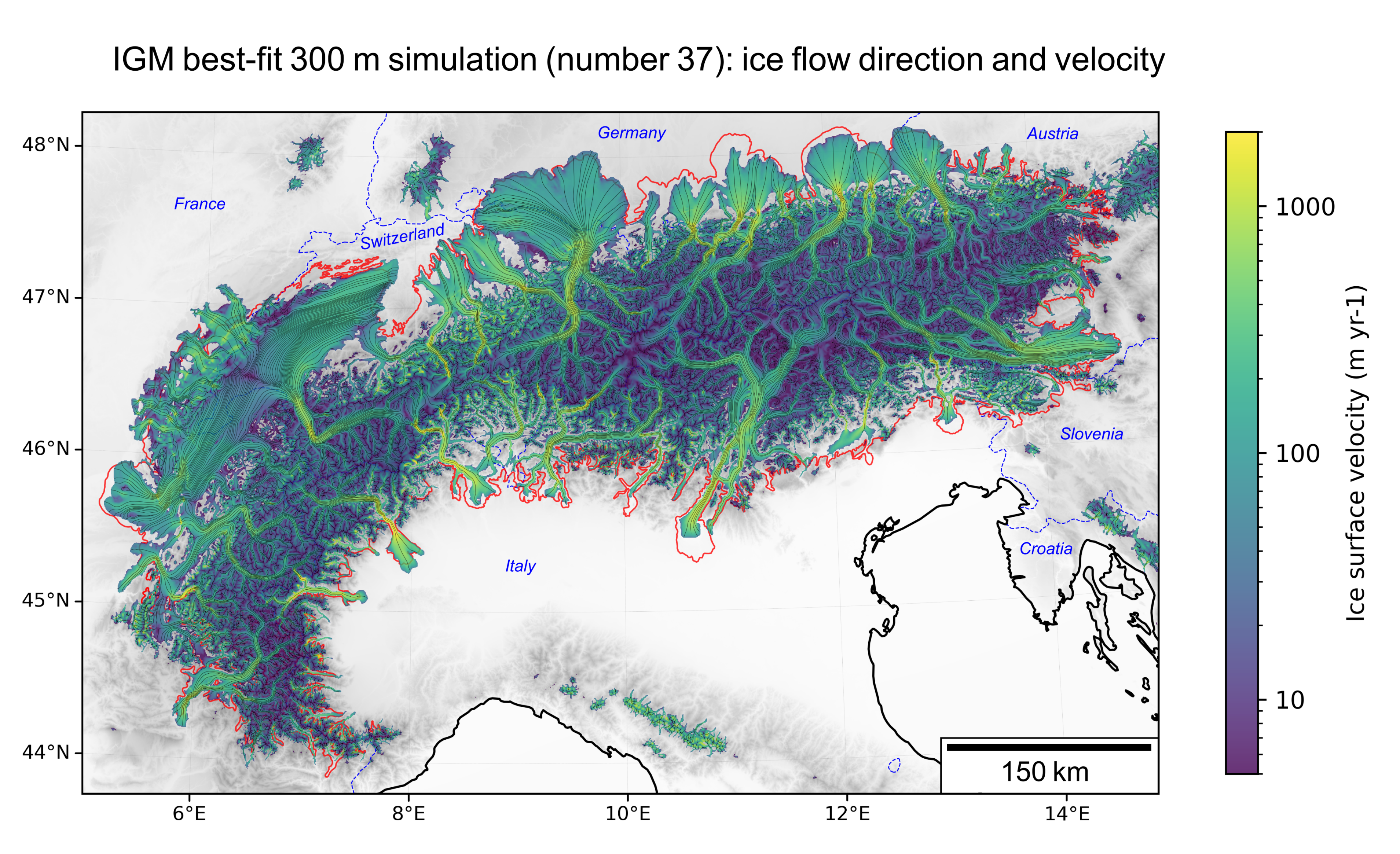
IGM best-fit 300 m simulation (number 37): ice flow direction and velocity
48°N
47°N
46°N
45°N
44°N
6°E
8°E
10°E
12°E
14°E
France
Switzerland
Germany
Austria
Slovenia
Croatia
Italy
150 km
1000
100
10
Ice surface velocity (m yr-1)

Supplementary Figure 20: Best-fit IGM 300 m simulation (number 37) results displayed by presenting its surface velocity field at the modelled LGM (24.8 ka) with superimposed static depth-averaged flow lines indicating both flow speed (line density) and trajectory (line direction). The empirical LGM outline of the AIF used in this study is shown in red, while country borders and coastlines are shown with dashed blue and thick black lines, respectively.

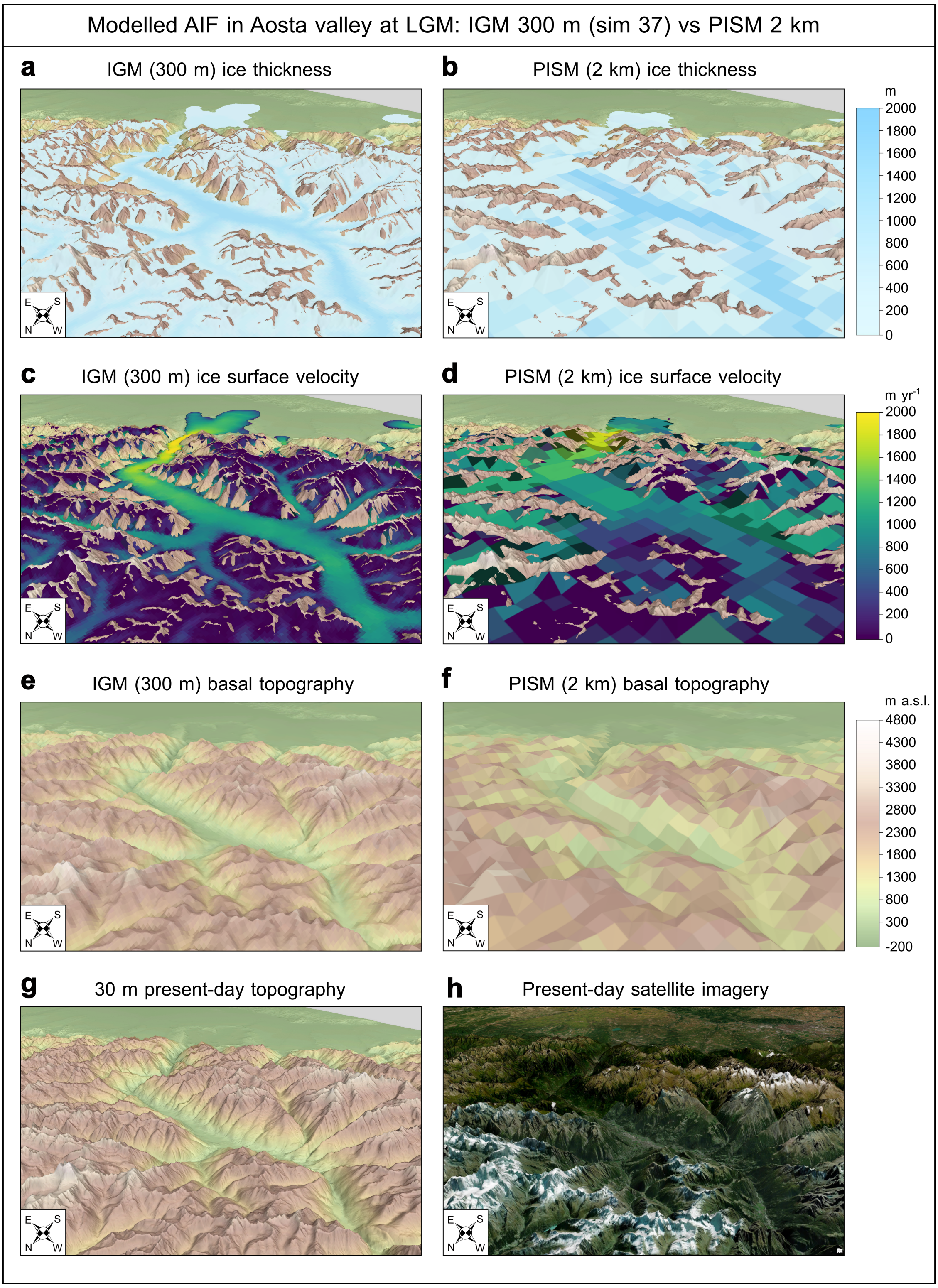

Supplementary Figure 21: Three-dimensional view of best-fit IGM 300 m simulation (number 37) compared with Jouvet *et al*. (2023)'s 2 km simulation displayed by showing modelled LGM ice thickness (panels **a**, **b**), ice surface velocity (panels **c**, **d**), and basal topography (panels **e**, **f**) fields in the main Aosta valley (looking towards the Southeast Alpine foreland). Ice thickness and velocity fields are plotted above a 30 m digital elevation model of the local topography (AW3D30 data), also shown in panel **g**. Panel **h** plots a satellite imagery of the same region (data from the Esri World imagery layer, source: Esri, Maxar, Earthstar Geographics, and the GIS User Community).

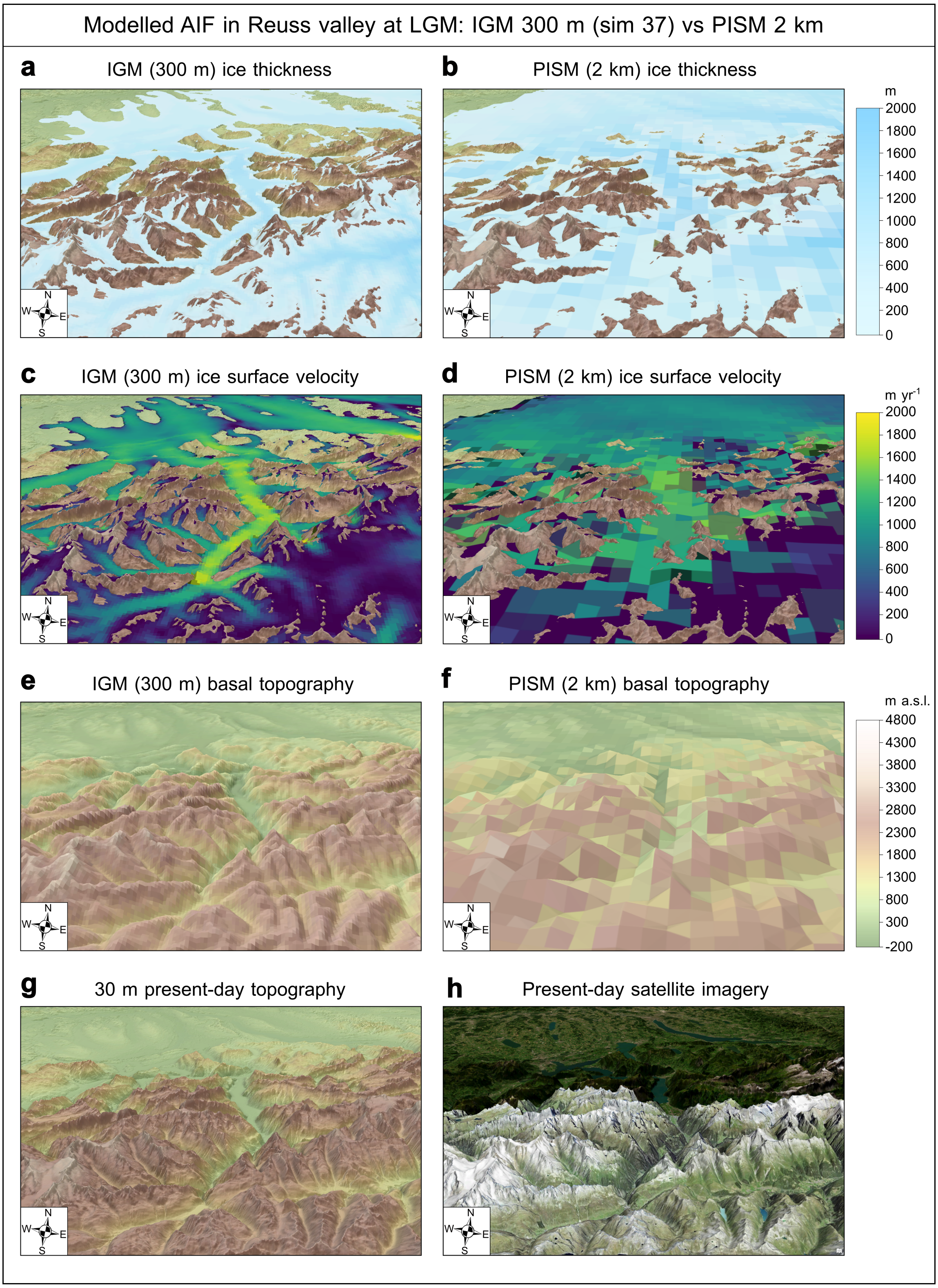

Supplementary Figure 22: Three-dimensional view of best-fit IGM 300 m simulation (number 37) compared with Jouvet *et al*. (2023)'s 2 km simulation displayed by showing modelled LGM ice thickness (panels **a**, **b**), ice surface velocity (panels **c**, **d**), and basal topography (panels **e**, **f**) fields in the main Reuss valley (looking North towards Alpine foreland). Ice thickness and velocity fields are plotted above a 30 m digital elevation model of the local topography (AW3D30 data), also shown in panel **g**. Panel **h** plots a satellite imagery of the same region (data from the Esri World imagery layer, source: Esri, Maxar, Earthstar Geographics, and the GIS User Community).
